# Sensory Processing Profiles in Depression: Evidence of Dynamic Changes and Clinical Correlates Over 3 Months

**DOI:** 10.1155/da/8238928

**Published:** 2026-09-04

**Authors:** Aude Paquet, Lucia Fiegl, Brigitte Plansont, Charlotte Bessaguet, Aude Gauthier, Benjamin Laplace

**Affiliations:** ^1^ Unité de Recherche et d’Innovation, Centre Hospitalier Esquirol, Limoges 87000, France; ^2^ INSERM, IRD, U1094 Tropical Neuroepidemiology, Institute of Epidemiology and Tropical Neurology, GEIST, Univ. Limoges, Limoges, France, inserm.fr; ^3^ HAVAE, UR 20217, Université de Limoges, Limoges F-87000, France, unilim.fr

**Keywords:** Adolescent/Adult Sensory Profile, clinical improvement, major depressive disorder, remission, sensory processing

## Abstract

**Background:**

Sensory processing difficulties are increasingly recognized in major depressive disorder (MDD), with patients often exhibiting heightened sensitivity, avoidance, and low registration. However, the temporal stability of these patterns—trait vs. state—remains unclear.

**Methods:**

In this longitudinal study, 45 adult inpatients with MDD were assessed using the Adolescent/Adult Sensory Profile (AASP) at admission and 3 months later; 34 completed both assessments. Clinical variables included depression (Hamilton Depression Rating Scale [HDRS‐17]), anxiety (Hamilton Anxiety Rating Scale [HARS]), anhedonia (Snaith‐Hamilton Pleasure Scale [SHAPS]), self‐esteem (Rosenberg Self‐Esteem Inventory [RSEI]), and psychomotor retardation (Clinical Outcomes in Routine Evaluation [CORE]). Nonparametric tests examined sensory changes and associations with clinical outcomes.

**Results:**

At follow‐up, 61.8% of participants showed clinical improvement and 44.1% achieved remission. Significant decreases in low registration and sensory sensitivity, along with increased sensation seeking, indicated that sensory processing is dynamic and partially state‐dependent. Sensory avoidance remained stable and was more pronounced in recurrent MDD. Patients with clinical improvement or remission had sensory profiles closer to normative ranges. Higher sensitivity and avoidance correlated with more severe depression, anxiety, and lower self‐esteem, while greater sensation seeking was associated with better outcomes.

**Conclusions:**

Sensory processing profiles in MDD are state‐dependent and linked to clinical changes. These findings support the relevance of sensory traits as potential clinical markers and therapeutic targets. Personalized interventions addressing sensory processing may contribute to improved treatment outcomes. Further longitudinal and multimodal research is needed to clarify their predictive value.

**Trial Registration:** ClinicalTrials.gov identifier: NCT05686668

## 1. Introduction

Sensory processing difficulties have recently emerged as an underestimated yet significant factor in a range of psychiatric disorders, particularly in major depressive disorder (MDD). International studies, notably those employing the Adolescent/Adult Sensory Profile (AASP), have consistently shown that individuals with MDD display distinct and atypical sensory profiles when compared to the general population, with increased prevalence of sensory sensitivity, sensory avoidance, and low registration [1–5]. This pattern is observed across diverse cultural and clinical contexts, suggesting that sensory processing anomalies may constitute a cross‐cutting dimension of depressive pathology. For instance, a multicentric study by Engel‐Yeger et al. [2] highlighted the impact of hypersensitivity on anxiety and sleep quality, while Paquet et al. [4] linked atypical sensory profiles with lower self‐esteem and greater psychomotor impairment. Beyond anhedonia and anxiety, extreme sensory processing patterns have been associated with broader affective dysregulation, including alexithymia and impaired quality of life, in patients with major affective disorders [6]. These findings suggest that atypical sensory processing may contribute to a heightened emotional vulnerability in MDD that extends beyond core depressive symptomatology.

Beyond these descriptive associations, recent investigations have questioned the temporal stability of sensory profiles in depression—specifically, whether these patterns reflect enduring “trait” characteristics or are modulated by clinical “state” [1]. To date, however, longitudinal studies evaluating changes in sensory processing alongside the course of depressive symptoms or recurrence are lacking. Addressing this gap is crucial: If sensory profiles prove dynamic and responsive to symptom evolution, they could serve both as clinical markers of progression and as potential targets for rehabilitative or environmental interventions.

Dunn’s Model of Sensory Processing, which underpins much of this research, provides an integrative framework relating individual neurological thresholds to behavioral self–regulation strategies [7]. This model situates individuals along two axes: neurological threshold (high versus low, indicating the amount of sensory input required to elicit a response) and self‐regulation strategy (active versus passive). The intersection of these axes defines four sensory processing patterns: low registration, sensation seeking, sensory sensitivity, and sensation avoiding. The AASP allows for structured quantification of these patterns, offering insights into the perceptual and behavioral responses to daily sensory events in psychiatric samples.

The clinical relevance of Dunn’s model in psychiatry has grown substantially in recent years, with evidence not only from depression but also from schizophrenia, bipolar disorder, anxiety disorders, and autism spectrum conditions [8]. In depression, the conceptual utility of the model lies in its capacity to shift clinical focus from symptom description to a nuanced understanding of how patients interact with environmental stimuli. This opens new directions for personalized care pathways, including environmental adjustments, occupational therapy interventions, and psychoeducation strategies.

In this context, clarifying the temporal directionality and clinical correlates of sensory processing profiles in depression is a necessary step, both for advancing pathophysiological understanding and for integrating sensory profiling into contemporary psychiatric practice.

Therefore, the main objective of this study was to compare the sensory processing profiles of adults with MDD at hospital admission and 3 months later, using the AASP. We also aimed to compare posthospitalization sensory profiles according to MDD recurrence, clinical improvement, and remission status. Finally, we examined whether key clinical variables (anxiety, depression, psychomotor retardation, anhedonia, and self‐esteem) differed according to sensory processing profiles.

Based on this background, we formulated the following a priori hypotheses: H1: Sensory processing patterns in adults with MDD would show significant changes between hospital admission and the 3‐month follow‐up, paralleling clinical improvement, suggesting that at least some sensory patterns function as state‐dependent markers rather than stable trait characteristics. H2: Patients with more extreme sensory processing patterns would display greater severity of depressive and anxiety symptoms, lower self‐esteem, and greater psychomotor retardation compared to patients with sensory profiles within normative ranges.

## 2. Methods

### 2.1. Study Design

This longitudinal prospective study was conducted in a naturalistic setting and included a first assessment at baseline (*T*0), followed by a further prospective evaluation 3 months later (*T*1).

### 2.2. Participants and Procedure

Participants with MDD (*n* = 45) were recruited at the Esquirol Hospital (Limoges, France) during hospitalization for MDD, between January 2022 and October 202. All patients were diagnosed with MDD by a psychiatrist according to the Diagnostic and Statistical Manual for Mental Disorders, 5th edition (DSM‐5).

Participants were between 18 and 65 years of age, without psychiatric comorbidities (e.g., bipolar disorder, substance use disorders other than tobacco, eating disorder, or obsessive–compulsive disorder), a known neurological history, neurodevelopmental disorder, or sensory or physical disability, and were able to answer questionnaires and understand French; moreover, they were not recruited from populations protected by French law.

Informed written consent was obtained from all participants, and the study was approved by a French committee for the Protection of the Persons, as required by French regulations. The study was conducted in accordance with the principles of the Declaration of Helsinki.

Sample size estimation was performed using 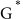
Power 3.1 software, based on a paired‐samples design to detect a difference in AASP quadrant scores within the same individuals between baseline (*T*0) and follow‐up (*T*1). Relying on published data and our previous findings in depressed samples [4], we expected a mean baseline of “low registration” (*T*0) score of 36.8 (SD  =  9.7) and a follow‐up (*T*1) mean of 30.29 (SD  =  6.25), corresponding to a clinically meaningful difference of 6.51 points. Assuming a correlation coefficient of 0.6 between baseline and follow‐up scores, we calculated an effect size (Cohen’s *d*) of 0.84. Using a two‐tailed test, an alpha risk of 0.05, and a power of 80%, the minimum sample required was 22 participants. However, we deliberately targeted the inclusion of 45 participants at baseline for the following reasons.

Expected attrition: Based on longitudinal follow‐up studies in MDD, attrition rates as high as 30% are commonly observed due to relapse, withdrawal, or loss to follow‐up.

Robustness for secondary and subgroup analyses: The protocol included planned comparisons according to clinical improvement, remission, and recurrence, as well as exploratory associations between sensory profiles and additional clinical variables. These analyses require larger sample sizes to achieve sufficient statistical power.

Generalizability: A larger sample improves result stability and extends external validity, especially important given the heterogeneity of depressive disorders.

This conservative approach was confirmed by our results: of the 45 included participants, 34 (76%) completed both assessments, validating our attrition estimate and ensuring adequate statistical power for both primary and secondary analyses.

### 2.3. Measurements

#### 2.3.1. The AASP

The AASP [7] is a validated 60‐item self‐report questionnaire designed to assess sensory processing patterns in daily life across six sensory domains (taste/smell, movement, vision, touch, activity level, and hearing). Items are rated on a five‐point Likert scale (“never” to “almost always”), with higher scores indicating greater endorsement of a given sensory experience. The AASP is grounded in Dunn’s [9] Model of Sensory Processing, which positions individuals along two orthogonal axes: neurological threshold (the amount of sensory input required to trigger a neurological response, ranging from high to low) and behavioral self‐regulation strategy (active or passive response to sensory input). The intersection of these two axes yields four clinically interpretable patterns: *low registration* (high threshold, passive response: reduced detection and registration of sensory input), *sensation seeking* (high threshold, active response: active search for increased sensory stimulation), *sensory sensitivity* (low threshold, passive response: heightened awareness of and distress from sensory input), and *sensation avoiding* (low threshold, active response: active behavioral withdrawal to limit sensory input). Subscale scores range from 15 to 75. Cut‐off values based on manual norms allow classification into five ordinal categories (much less than most people, less than most, similar to most, more than most, and much more than most), facilitating both continuous and categorical analyses.

The French version of the AASP was administered, as made available by the publisher (Pearson, France). To date, although the French translation is routinely used in clinical and research settings and referenced in several French studies involving clinical populations (e.g., [4, 10]), a comprehensive psychometric validation of the French version—including reliability coefficients, factor structure, and convergent validity—has not yet been formally published. As such, the psychometric properties referenced for this instrument are drawn from the original English‐language validation studies. This limitation should be considered in interpreting the results. Cut‐off values are based on manual norms and allow classification into five ordinal categories (much less than most people, less than most, similar to most, more than most, and much more than most), facilitating both continuous and categorical analyses.

#### 2.3.2. Hamilton Depression Rating Scale (HDRS‐17)

The HDRS‐17 [11, 12] is a clinician‐administered scale that assesses the severity of depressive symptoms over the previous week. It consists of 17 items, each rated on a scale from 0 to 2 or 0–4, with total scores ranging from 0 to 52. Scores are interpreted as follows: <7 indicates no depression; 8–16, mild depression; 17–23, moderate depression; and >24, severe depression [13]. Clinical improvement at follow‐up (*T*1) was defined as a ≥50% reduction from baseline (*T*0), while remission corresponded to an HDRS‐17 score ≤ 8 at *T*1, in line with established criteria.

#### 2.3.3. Hamilton Anxiety Rating Scale (HARS)

The HARS [14] is a clinician‐administered 14‐item scale measuring the severity of anxiety symptoms, including both psychic and somatic domains. Each item is rated on a 5‐point scale (0  =  not present to 4  =  severe), providing a total score from 0 to 56. A score ≥ 20 was considered indicative of clinically significant anxiety. The HARS has demonstrated good reliability and validity in adult clinical populations.

#### 2.3.4. Snaith‐Hamilton Pleasure Scale (SHAPS)

The SHAPS [15] is a self‐administered questionnaire designed to evaluate hedonic tone and anhedonia. It comprises 14 items covering four domains of pleasurable response. Each item was scored using four response options (strongly agree, agree, disagree, and strongly disagree), with a total score from 0 to 14; higher scores reflect greater anhedonia. The SHAPS has established validity and reliability for assessing hedonic capacity in adults with MDD [16].

#### 2.3.5. Rosenberg Self‐Esteem Inventory (RSEI)

Self‐esteem was measured using the RSEI [17], a 10‐item self‐report questionnaire assessing global self‐worth. Each statement is rated on a four‐point Likert scale, yielding a total score between 10 and 40. Cut‐off scores are as follows: <25, very low; 25–30, low; 31–34, medium; 35–39, high; and >39, very high self‐esteem. The RSEI has robust psychometric properties in psychiatric populations [18].

#### 2.3.6. Clinical Outcomes in Routine Evaluation (CORE)

Psychomotor retardation was assessed with the CORE scale [19], an 18‐item clinician–rated instrument focusing on observable psychomotor symptoms during the clinical interview. Scores range from 0 to 54, with higher scores reflecting greater psychomotor impairment. A cut‐off of 8 was used to identify melancholic depression [20]. The CORE has shown good inter‐rater reliability and validity for detecting melancholic features in depression [21].

### 2.4. Procedure

All assessments were conducted individually by a trained therapist, using standardized, paper‐and‐pencil questionnaires. At both time points (*T*0 and *T*1), the following instruments were administered in the same fixed order: HDRS‐17 and HARS, followed by the SHAPS and the RSEI, then the AASP. This administration sequence was chosen to maintain consistency and minimize order effects. All questionnaires were completed under the experimenter’s supervision, ensuring participant comprehension and reducing missing data.

### 2.5. Statistics

Statistical analyses were conducted using Statistical Package for Social Science (SPSS) 21 commercial software for Windows on PC. Categorical variables were presented as percentages, while quantitative variables were presented as medians and interquartile ranges (IQRs). Nonparametric tests were used. Comparisons were performed using the nonparametric Mann–Whitney test between groups or Wilcoxon test between *T*0 and *T*1. For multiple comparisons of quantitative variables, the Kruskal–Wallis test was used. Post hoc Mann–Whitney tests with Bonferroni correction were conducted to compare one group to others (*p* = 0.025). For these comparisons, clinical scale scores were used. For other statistical analyses, the significance threshold was 5%.

## 3. Results

Forty‐five participants were included in the study, of whom 34 completed both assessments at *T*0 and *T*1. Reasons why participants did not complete the assessment at *T*1 were as follows: one death, one withdrawal of consent, five lost to follow‐up, and four who were unable to attend the second visit. Analyses were conducted using the 34 participants who attended both visits.

### 3.1. Clinical Profile

The characteristics of the MDD participants and their clinical profiles were presented in Table 1. A clinical improvement between *T*1 and *T*0 was observed in 61.8 % of the participants (*n* = 21). Clinical scale scores were significantly improved, indicating clinical improvement (Table 1). Based on their HDRS‐17 score, 15 participants were in remission at *T*1 (44.1%).

**Table 1:** Clinical characteristics of major depressive disorder participants (MDD) at baseline (*T*0) and 3 months (*T*1).

Variables	*T*0 (*n* = 34)	*T*1 (*n* = 34)	*p*‐Value
Age (years) (median, IQR)	47 (36–56)		—
Sex (M/F)	13/21		—
MDD isolated/recurrent (%)	61.8% (21)/38.2% (13)		—
Treatment (%)			
Antidepressant	100%	97%	—
Anxiolytic	76.5%	45.5%	—
Hypnotic	29.4%	15.2%	—
Repetitive transcranial magnetic stimulation (rTMS)	0%	0%	—
HDRS‐17 score (median, IQR)	22 (18.75–25.25)	8.5 (3.75–8.5)	*Z* = −4.84, *p* < 0.001
HARS score (median, IQR)	22.5 (18.75–26)	11 (5.75–17)	*Z* = −4.56, *p* < 0.001
CORE total score (median, IQR)	3 (0–7.25)	1 (0–2)	*Z* = −3.52, *p* < 0.001
SHAPS score (median, IQR)	5.5 (3.75–8)	2 (0–5.5)	*Z* = −3.29, *p* < 0.001
RSEI score (median, IQR)	18 (15–22.5)	24 (19.75–31)	*Z* = 4.04, *p* < 0.001

There were differences in HDRS‐17 and HARS scores according to sex at *T*0. Women had more depressive symptoms (*Z* = −2.26 and *p* = 0.024) and more anxiety symptoms (*Z* = −2.70 and *p* = 0.006). These differences were not observed at *T*1.

Clinical characteristics of the MDD participants according to MDD recurrence, clinical improvement, and remission rate were presented in Table 2.

**Table 2:** Clinical characteristics of participants according to the recurrence (*R*) or isolated (*I*) major depressive disorder (a), the clinical improvement (CI+) or not (CI−) (b), and the remission (*R*+) or not (*R*−) (c).

a.				
Variables	*R* (*n* = 13)		*I* (*n* = 21)	*p*‐Value
Age (years) (median, IQR)	53 (43–56)		47 (29–53)	ns
Gender (M/F)	1/12		12/9	*p* = 0.005
HDRS‐17 score (median, IQR)	8 (2–15)		9 (4–14)	ns
HARS score	10 (6–15)		12 (7–19)	ns
CORE total score	1 (0–2)		0 (0–2)	ns
SHAPS score	4 (0–5)		2 (0–4)	ns
RSEI score	24 (20–31)		24 (20–29)	ns

**b.**				

**Variables**	**CI+ (*n* = 21)**		**CI− (*n* = 13)**	***p*-Value**

Age (years) (median, IQR)	48 (43–56)		44 (33–53)	ns
Gender (M/F)	9/12		4/9	ns
HDRS‐17 score (median, IQR)	4 (2–8)		16 (14–20)	*Z* = −4.35, *p* < 0.001
HARS score	6 (4–10)		17 (14–23)	*Z* = −3.73, *p* < 0.001
CORE total score	0 (0–1)		2 (1–2)	*Z* = −2.35, *p* < 0.001
SHAPS score	1 (0–2)		7 (4–8)	*Z* = −4.10, *p* = 0.027
RSEI score	29 (24–34)		19 (16–21)	*Z* = −3.55, *p* < 0.001

**c.**				

**Variables**	***R*+ (*n* = 15)**		***R*− (*n* = 19)**	***p*-Value**

Age (years) (median, IQR)	47 (33–60)		48 (39–54)	ns
Gender (M/F)	7/8		6/13	ns
HDRS‐17 score (median, IQR)	3 (1–4)		14 (11–16)	*Z* = −4.96, *p* < 0.001
HARS score	5 (2–6)		16 (13–22)	*Z* = −4.69, *p* < 0.001
CORE total score	0 (0–2)		1 (0–2)	ns
SHAPS score	0 (0–2)		4 (2–8)	*Z* = −3.35, *p* < 0.001
RSEI score	31 (26–37)		21 (17–24)	*Z* = −3.91, *p* = 0.001

### 3.2. Evolution of Sensory Processing Patterns

**Figure 1:**
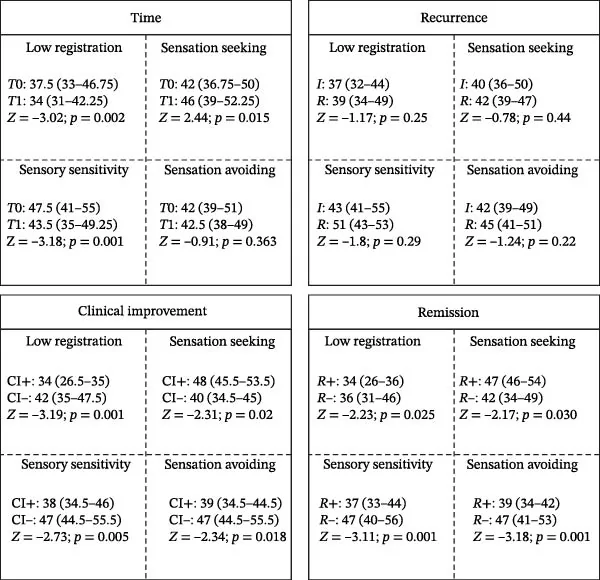
Scores (median, IQR) in each sensory processing pattern for major depressive disorder (MDD) according to time, MDD recurrence, clinical improvement, and remission. *T*0: baseline; *T*1: 3 months; *I*: isolated MDD; *R*: recurrent MDD; CI+: clinical improvement; CI−: no clinical improvement; R+: remission; R−: no remission.

The scores of the four AASP patterns according to time, MDD recurrence, clinical improvement, and remission are reported in Figure 1. Sensory processing evolution according to time was different for three AASP patterns between *T*0 and *T*1. Scores in *low registration* and *sensory sensitivity* decreased significantly at *T*1, whereas *sensation seeking* increased at *T*1.

For all AASP patterns, sensory processing after clinical improvement differed between patients with and without clinical improvement and between patients with and without remission. Patients with clinical improvement and patients with remission had lower scores on *low registration*, *sensory sensitivity*, and *sensation avoiding* and higher scores on *sensation seeking* than those without improvement or remission.

There was no difference in AASP patterns between participants with recurrent MDD or isolated MDD.

**Figure 2:**
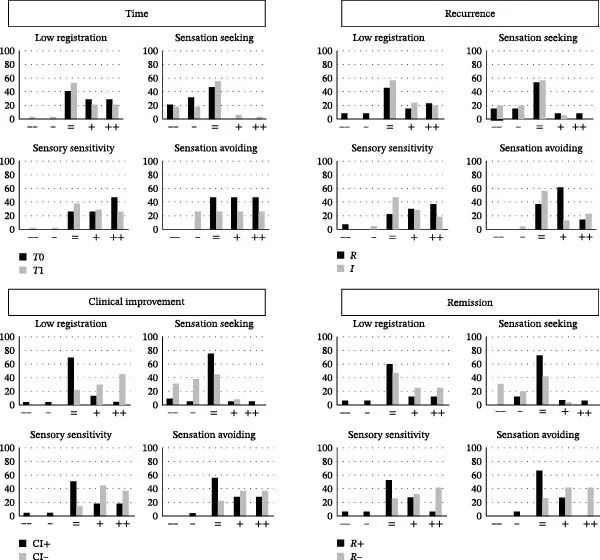
Percentage of participants who were found in the normal ( = ) and abnormal (− −; −; +; ++) range of the AASP according to time, MDD recurrence, clinical improvement, and remission. *T*0: baseline; *T*1: 3 months; *I*: isolated MDD; *R*: recurrent MDD; CI+: clinical improvement; CI−: no clinical improvement; *R*+: remission; *R*−: no remission.

Figure 2 summarized the percentage of participants who were found in the normal ( = ) and abnormal (− −; −; +; ++) range of the AASP. Based on the AASP norms, patients with a *low registration* scoring similar to most people ( = ) increased at *T*1 compared to *T*0 (from 41% at *T*0 to 53% at *T*1), patients with *sensation seeking* scoring similar to most people increased at *T*1 (from 47% at *T*0 to 56% at *T*1), patients with *sensory sensitivity* scoring similar to most people increased at *T*1 (from 26% at *T*0 to 38% at *T*1), and patients with *sensation avoiding* scoring similar to most people also increased at *T*1 (from 41% at *T*0 to 44% at *T*1). Patients with extreme scores remained high.

Although there was no difference in AASP scores between isolated and recurrent MDD (Figure 1), the distribution of scores in *sensation avoiding* quadrant was significantly different between patients with isolated or recurrent MDD (*p* = 0.029). Profile of sensation avoiding was more extreme among patients with recurrent MDD.

Regarding clinical improvement, the distribution of scores in *low registration* (*p* = 0.004) and *sensation seeking* (*p* = 0.05) was significantly different between patients with and without clinical improvement. Patients with clinical improvement had more normalized scores.

Regarding remission, the distribution of scores in *sensation seeking* (*p* = 0.047), *sensory sensitivity* (*p* = 0.049), and *sensation avoiding* (*p* = 0.007) was significantly different between patients in remission or not. Patients in remission at *T*1 had more normalized scores.

### 3.3. Variations in Clinical Data Based on Sensory Processing Patterns

To compare scores on the clinical scales (anxiety, depression, psychomotor impairment, anhedonia, and self‐esteem) based on sensory processing patterns at two times, we recoded the results for each participant into three categories: less than most people (<MP), similar to most people ( = MP), and more than most people (>MP). Significant differences between the profiles in each quadrant (*low registration*, *sensation seeking*, *sensory sensitivity*, and *sensation avoiding*) based on the clinical data are presented in Table 3.

**Table 3:** Difference in clinical data, according to the three profiles (<MP, = MP, and >MP) in each quadrant (low registration, sensation seeking, sensory sensitivity, and sensation avoiding) of the Adolescent/Adult Sensory Profile.

Clinical variables	Low registration				Sensation seeking			
	<MP	= MP	>MP	H; p	<MP	= MP	>MP	H; p
Baseline								
HDRS‐17	—	22 (19–23)	22 (19–26)	ns	22 (20–24)	22 (19–26)	—	ns
HARS	—	21 (18–23)	24 (21–26)	ns	24 (21–26)	22 (19–26)	—	ns
CORE	—	2 (0–5)	4 (2–9)	ns	3 (0–5)	4 (0–8)	—	ns
SHAPS	—	4 (3–5)	7 (5–8)	ns	8 (6–9)	4 (3–5)	—	6.80; 0.009
RSEI	—	19 (17–23)	18 (15–23)	ns	17 (14–24)	19 (18–21)	—	ns
At 3 months								
HDRS‐17	1 (1–1)	8 (2–13)	14 (7–20)	9.38; 0.009	14 (9–19)	7 (2–13)	2 (0–14)	8.56; 0.014
HARS	2 (1–2)	10 (4–14)	16 (6–23)	8.25; 0.016	14 (13–23)	6 (4–15)	7 (1–17)	5.69; 0.058
CORE	0 (0–0)	0 (0–2)	2 (1–4)	ns	2 (1–2)	0 (0–2)	1 (0–6)	ns
SHAPS	0 (0–0)	2 (0–4)	4 (1–8)	ns	6 (4–8)	1 (0–4)	1 (0–5)	7.28; 0.026
RSEI	38 (37–39)	26 (22–31)	21 (19–26)	7.06; 0.029	19 (17–25)	26 (23–34)	31 (19–40)	8.24; 0.016

Clinical variables	Sensory sensitivity				Sensation avoiding			
	<MP	= MP	>MP	H; p	<MP	= MP	>MP	H; p
Baseline								
HDRS‐17	—	20 (19–22)	22 (20–26)	ns	—	20 (18–22)	22 (22–27)	4.92; 0.027
HARS	—	21 (18–22)	24 (21–26)	ns	—	21 (17–23)	21 (21–26)	ns
CORE	—	1 (0–4)	4 (1–8)	ns	—	2 (0–4)	5 (2–8)	4.40; 0.036
SHAPS	—	4 (3–4)	6 (4–8)	ns	—	4 (2–7)	6 (4–9)	ns
RSEI	—	20 (19–24)	17 (15–21)	3.62; 0.057	—	21 (19–24)	16 (15–19)	9.90; 0.002
At 3 months								
HDRS‐17	2 (1–2)	4 (2–11)	14 (7–16)	7.92; 0.019	2 (2–2)	4 (2–11)	14 (8–15)	5.96; 0.051
HARS	2 (1–3)	6 (5–13)	14 (9–19)	7.55; 0.023	3 (3–3)	6 (4–13)	14 (9–19)	7.39; 0.025
CORE	0 (0–0)	0 (0–2)	1 (0–2)	ns	0 (0–0)	0 (0–2)	1 (1–2)	ns
SHAPS	1 (0–1)	2 (0–4)	4 (1–7)	ns	1 (1–1)	0 (0–2)	4 (1–7)	ns
RSEI	34 (29–39)	31 (24–36)	21 (17–26)	11.35; 0.003	29 (29–29)	31 (22–37)	23 (17–26)	7.59; 0.022

Abbreviation: MP, most people.

#### 3.3.1. Low Registration

Based on the *low registration* profile, analyses of clinical scale scores showed a significant effect of group (<MP, = MP, and >MP) at 3 months, on depression, anxiety, and self‐esteem scales. Post hoc Mann–Whitney *U* tests showed that very hyposensitive people (significantly higher scores on low registration) had higher depression scores (*Z* = −2.29 and *p* = 0.022) than those with *low registration* scores in the norm ( = MP).

#### 3.3.2. Sensation Seeking

Based on the *sensation seeking* profile, analyses of clinical scale scores showed a significant effect of group (<MP, = MP, and >MP) at 3 months, on depression, anxiety, anhedonia, and self‐esteem scales. Post hoc *U* tests showed that the people who had low *sensation seeking* scores (<MP) had higher depression scores (*Z* = −2.73 and *p* = 0.006), higher anxiety scores (*Z* = −2.36 and *p* = 0.018), higher anhedonia scores (*Z* = −2.62 and *p* = 0.008), and lower self‐esteem (*Z* = −2.76 and *p* = 0.005) than those with *sensation seeking* scores in the norm ( = MP).

#### 3.3.3. Sensory Sensitivity

Based on the *sensory sensitivity* profile, analyses of clinical scale scores showed a significant effect of group (<MP, = MP, and >MP) at 3 months on depression, anxiety, and self‐esteem. Post hoc *U* tests showed that the people who had high *sensory sensitivity* (>MP) had lower self‐esteem (*Z* = −2.94 and *p* = 0.003) than those with *sensation sensitivity* scores in the norm ( = MP).

#### 3.3.4. Sensation Avoiding

Based on the *sensation avoiding* profile, analyses of clinical scale scores showed a significant effect of group (<MP, = MP, and >MP) at 3 months, on anxiety and self‐esteem. Post hoc *U* tests showed that people with high *sensory avoiding* had higher anxiety levels (*Z* = −2.43 and *p* = 0.015) and lower self‐esteem (*Z* = −2.59 and *p* = 0.008) than those with *sensation avoiding* scores in the norm ( = MP).

## 4. Discussion

### 4.1. Sensory Processing Profiles and Clinical Evolution

The results of this study confirm the existence of distinct sensory processing profiles among patients with MDD and suggest that these profiles evolve in conjunction with clinical improvement. At baseline, the sensory processing patterns observed mirrored those reported in our previous work [4]. Patients exhibited abnormally low sensory seeking, heightened sensory sensitivity, increased sensory avoidance, and a reduced capacity for registering sensory information.

Our data demonstrated a significant decrease in low registration and sensory sensitivity scores 3 months after hospitalization, whereas sensory seeking normalized and sensory avoidance remained unchanged. This reduction in low registration and sensory sensitivity has also been described by Engel‐Yeger et al. [3], though at a 6‐month interval. Notably, their cohort consisted of stabilized outpatients, in contrast to our sample of inpatients with severe symptomatology, indicating that pronounced symptomology may render sensory profile changes detectable more rapidly.

According to Dunn’s model, this shift suggests that patients adopt more active behavioral strategies 3 months posthospitalization compared to the acute phase of MDD. Improvements in AASP scores paralleled clinical improvement and remission across all sensory processing patterns. While Engel‐Yeger et al. [1] proposed that extreme sensory profiles may serve as stable “trait” markers in affective disorders, our findings indicate that three patterns varied over time and four patterns changed with symptom evolution. Thus, sensory profiles in this context appear to be more state‐dependent, reflecting responses to reduced anxiety, mood improvement, and alleviation of depressive symptoms.

Based on these findings, clinicians should pay close attention to sensory information processing in depression, as it may serve as an indicator of clinical improvement. Larger‐scale studies are needed to determine whether sensory profiles can predict treatment response. Moreover, the significance of sensory profile modifications within the symptomatology of MDD remains to be clarified. Only longitudinal studies can establish whether sensory processing difficulties predate the onset of MDD.

### 4.2. Recurrence of MDD and Sensory Profiles

The absence of significant differences in AASP scores between patients with recurrent and first‐episode MDD suggests that sensory profiles are not specific markers of recurrence. Nevertheless, patients with recurrent MDD exhibited more extreme sensory avoidance profiles, and this pattern remained stable over time. In their investigation of predictors for antidepressant response, Engel‐Yeger et al. [3] reported that sensory avoidance accounted for 14% and 5% of the variance in depression after 3 and 6 months of medication, respectively. This association may imply that sensory avoidance either fosters chronicity or represents an alteration induced by chronic depression.

Serafini et al. [5] found that a longer episode duration was associated with distinct sensory processing depending on mood disorder subtype: in unipolar patients, chronicity correlated with greater sensory registration and less avoidance, while bipolar patients showed heightened sensory sensitivity and avoidance but reduced registration. These findings highlight the complex, biphasic nature of sensory processing in relation to illness progression.

### 4.3. Sensory Processing and Clinical Variables

Low self‐esteem, depressive, and anxiety symptoms remained higher at 3 months in participants with passive behavioral strategies (low registration > mean population and sensory sensitivity > mean population), as well as in those employing more active avoidance to manage sensations (sensory sensitivity > mean population). Conversely, participants using active coping strategies such as increased sensation seeking (sensory seeking > mean population) or normative sensory sensitivity (sensory sensitivity = mean population) had higher self‐esteem and lower depressive, anxiety, and anhedonia scores at 3 months.

A recent systematic review suggested that high sensory sensitivity can negatively impact quality of life, leading to fluctuations in well‐being, daily functioning, and health status [22]. Pérez‐Chacón et al. [23] found that individuals with elevated sensitivity reported lower health‐related quality of life, greater introversion, and a tendency toward maladaptive coping strategies, corroborating other studies linking extreme sensory sensitivities to reduced quality of life [1, 24, 25]. Research on sensory processing sensitivity (a personality trait characterized by elaborate information processing and high sensitivity) per Aron and Aron [26] indicates a positive association with negative psychological symptoms. Highly sensitive individuals often avoid emotions [6] and social situations [23]. Yano et al. [27] showed that high sensory sensitivity correlated with higher depression scores among students with low sense of coherence. The link between sensory processing sensitivity, depression, and anxiety may partially rest on emotion regulation strategies that modulate how individuals with depression process sensory information.

### 4.4. Clinical Perspectives

These findings open relevant avenues for integrating sensory processing assessment into clinical management of MDD. Sensory information processing difficulties are not confined to conditions such as autism or neurodevelopmental disorders but are also present in psychiatric illnesses and should be considered both in symptom assessment and their impact on patients’ everyday life.

Taking into account patients’ sensory profiles could help tailor therapeutic interventions. Sensory processing information can be incorporated into psychoeducational programs for patients with depression. Objectives might include the following:

- Understanding sensory processing and related behavioral strategies in daily life.
- Identifying sensory situations that induce discomfort or stress.
- Learning to manage sensory sensitivities through protective strategies, sensory reinforcement, soothing techniques, or structured environments, aiming to modify or reduce avoidance behaviors, especially social withdrawal.

For example, patients with sensory avoidance profiles could benefit from interventions to gradually increase sensory tolerance, while those with low sensory seeking might profit from activities encouraging sensory engagement.

Virtual reality systems provide controlled, immersive environments with modifiable sensory stimuli [28, 29]. These allow for gradual sensory stimulation and incorporation of various sensory inputs in different contexts, with real‐time behavioral and neurophysiological feedback guiding incremental exposure until appropriate responses develop [30]. Such systems could represent an innovative clinical tool for patients with depression and sensory processing difficulties.

Other techniques like repetitive transcranial magnetic stimulation (rTMS) and tDCS hold promise for studying and potentially treating sensory processing disorders, as they can modulate neural network activity involved in top‐down regulation [31] and assess information flow between posterior (sensory) and prefrontal (executive) brain regions [32]. Their already established utility in depression [33–35] invites future research into their impact on sensory processing in MDD.

### 4.5. Limitations and Perspectives

This study has several limitations that warrant consideration. First, the relatively small sample size limits the statistical power of subgroup analyses and limits generalizability to other clinical settings, particularly outpatient settings. Second, although our findings are consistent with those of our previous study including a healthy control group [4], the absence of such a group in the present study limits direct comparison with normative data. The inclusion of a control group would have strengthened the interpretation of sensory profiles by providing a contemporaneous reference framework. Third, relying exclusively on self‐reporting, including the AASP, may introduce subjectivity and reporting biases. Future studies should incorporate objective psychophysical or neurophysiological measures of sensory processing to complement self‐report data. Fourth, the French version of the AASP has not yet undergone formal independent psychometric validation, which includes the publication of reliability coefficients, factor structure, and convergent validity. Although it is routinely used in French clinical and research settings, this limits the interpretability of subscale scores compared to the original English‐language version. Fifth, details of pharmacological treatment, such as type, dose, and duration of antidepressant therapy, could not be systematically integrated into the analysis, in contrast to studies such as Engel‐Yeger et al. that specifically examined the interaction between sensory profiles and antidepressant response. Addressing this issue in future research could improve the accuracy and scope of the results.

Sixth, while the 3‐month follow‐up period is sufficient to capture short‐term dynamics, it precludes conclusions regarding the persistence or potential fluctuation of sensory processing profiles over longer timeframes. Future longitudinal cohort studies, with an extended follow‐up period, would be valuable to clarify whether sensory processing patterns represent stable trait markers of vulnerability to MDD or rather state markers that fluctuate with the clinical course. Such studies could also explore whether sensory processing assessments have predictive value for the recurrence or chronicity of depressive episodes.

Seventh, participant recruitment partly coincided with the COVID‐19 pandemic. Psychosocial stressors associated with the pandemic may have influenced the sensory reactivity and emotional vulnerability of hospitalized patients. This represents a contextual variable that could not be accounted for in our analyses [36].

Despite these limitations, our findings offer new insights into the dynamics of sensory processing in depression and underscore the need for multimodal, long‐term, and integrative approaches to better characterize these sensory dimensions in psychiatric populations.

## 5. Conclusion

This longitudinal study provides novel evidence that sensory processing profiles in adults with MDD are dynamic and partly state‐dependent, exhibiting significant changes over a 3‐month period, paralleling clinical improvement and remission.

More specifically, three of the four sensory patterns (low registration, sensory sensitivity, and sensation seeking) normalized significantly with clinical improvement, suggesting they function as state markers responsive to treatment. Sensation avoiding, by contrast, remained stable regardless of clinical trajectory and was more pronounced in patients with recurrent MDD, pointing to its potential role as a trait‐like vulnerability marker. At the clinical level, sensory sensitivity and sensation avoiding were robustly associated with greater depression severity, anxiety, and lower self‐esteem, while improved sensation seeking was linked to better outcomes at follow‐up.

Taken together, these findings carry three main implications: sensory processing patterns are dynamic and partly state‐dependent rather than solely stable trait markers; specific patterns carry distinct prognostic implications; and AASP‐based sensory profiling may complement standard symptom scales in routine psychiatric assessment, particularly to identify patients at risk of impeded recovery or chronicity.

From a therapeutic standpoint, these findings open preliminary avenues for future research. The trait‐like stability of sensation avoiding in recurrent MDD raises the question of whether targeting sensory processing directly, through bottom‐up approaches such as sensory‐based occupational therapy or psychomotor interventions, could complement standard treatment and support recovery. Empirical validation through interventional studies is needed to explore these possibilities.

However, the modest sample size and reliance on self‐report instruments call for cautious interpretation. Future research with larger cohorts, extended follow‐up, and multimodal sensory assessments is needed to deepen understanding of sensory processing alterations and their prognostic significance in depressive disorders.

## Author Contributions

**Aude Paquet**: conceptualization, methodology, funding acquisition, investigation, writing – original draft preparation. **Lucia Fiegl**: investigation, writing – reviewing and editing. **Brigitte Plansont**, **Charlotte Bessaguet, and Aude Gauthier**: resources. **Benjamin Laplace**: investigation, formal analysis, supervision, writing – reviewing and editing.

## Funding

This study was supported through funding provided by the Fondation pour la Recherche en Psychomotricité et Maladie de Civilisation (Fondation de France). This financial support made it possible to collect the data, write the report, and publish it.

## Ethics Statement

The study was approved by the French Committee for the Protection of the Persons (Est 1), according to French regulations. The study followed the principles of the Declaration of Helsinki.

## Consent

Informed written consent was obtained from all participants.

## Conflicts of Interest

The authors declare no conflicts of interest.

## Data Availability

Data may be available upon request.
